# Cytomegalovirus Replication Kinetics in Solid Organ Transplant Recipients Managed by Preemptive Therapy

**DOI:** 10.1111/j.1600-6143.2012.04087.x

**Published:** 2012-09

**Authors:** S F Atabani, C Smith, C Atkinson, R W Aldridge, M Rodriguez-Perálvarez, N Rolando, M Harber, G Jones, A O’Riordan, A K Burroughs, D Thorburn, J O’Beirne, R S B Milne, V C Emery, P D Griffiths

**Affiliations:** a Division of Infection and Immunity, Centre for VirologyLondon, UK; b Department of Infection & Population HealthLondon, UK; c Centre for Infectious Disease EpidemiologyLondon, UK; d Sheila Sherlock Liver Centre, Royal Free NHS TrustLondon, UK; e UCL Kidney and Urology Centre, Royal Free NHS Trust & UCL Medical SchoolLondon, UK

**Keywords:** Allograft, cytomegalovirus replication, liver, preemptive management, renal

## Abstract

After allotransplantation, cytomegalovirus (CMV) may be transmitted from the donor organ, giving rise to primary infection in a CMV negative recipient or reinfection in one who is CMV positive. In addition, latent CMV may reactivate in a CMV positive recipient. In this study, serial blood samples from 689 kidney or liver transplant recipients were tested for CMV DNA by quantitative PCR. CMV was managed using preemptive antiviral therapy and no patient received antiviral prophylaxis. Dynamic and quantitative measures of viremia and treatment were assessed. Median peak viral load, duration of viremia and duration of treatment were highest during primary infection, followed by reinfection then reactivation. In patients who experienced a second episode of viremia, the viral replication rate was significantly slower than in the first episode. Our data provide a clear demonstration of the immune control of CMV in immunosuppressed patients and emphasize the effectiveness of the preemptive approach for prevention of CMV syndrome and end organ disease. Overall, our findings provide quantitative biomarkers which can be used in pharmacodynamic assessments of the ability of novel CMV vaccines or antiviral drugs to reduce or even interrupt such transmission.

## Introduction

Human cytomegalovirus (CMV) is an important opportunistic pathogen and the cause of significant morbidity and some mortality among patients undergoing hematopoietic stem cell or solid organ transplantation (SOT; Ref. 1). Natural history studies have shown that viral replication kinetics, peak and cumulative viral load in whole blood posttransplant correlate with the development of CMV end-organ disease (EOD; Refs. 2,3).

In many transplant centers, the mainstay of CMV management of high-risk patients posttransplant is the prophylactic use of antiviral medication, such as ganciclovir or its prodrug valganciclovir, for the initial 100 days posttransplant. Extended prophylaxis (200 days) gives improved control of CMV (4), however, patients still remain at risk of late onset syndrome/disease once prophylaxis is stopped and some cases have strains of CMV which are resistant to ganciclovir (5).

In an alternative approach, regular monitoring of CMV DNA in blood by quantitative real-time PCR (qPCR) guides preemptive therapeutic intervention for CMV disease prevention (6). Randomized controlled trials show that prophylaxis and preemptive therapy are both effective strategies for controlling CMV and preventing EOD after renal and liver transplantation (7–9). Our transplant center has routinely used the strategy of qPCR-driven preemptive therapy to prevent CMV EOD since 2002.

This study describes the natural history of CMV infection postrenal or liver transplantation in the era of qPCR guided preemptive therapy and documents the effectiveness of this approach for preventing CMV syndrome and EOD. It highlights the differences in CMV replication kinetics that continue to exist between and within different patient populations and provides important data on replication parameters that will guide the evaluation of new antiviral drugs and candidate vaccines for the prevention of CMV infection posttransplant.

## Materials and Methods

### Patients

All liver (374 patients) and renal (497 patients) transplants performed and/or followed up at the Royal Free Hospital, London, between July 2002 and the end of January 2010, were identified from the transplant databases. Patients were excluded if their CMV serostatus, or that of their donor, was unknown, if they had participated in the active arms of other ongoing studies including an experimental CMV vaccine study reported elsewhere (10), if they had received valganciclovir prophylaxis or if they received a multi-organ transplant. (Excluded patients did not differ from the study cohort with respect to age, underlying disease, or specific immunosuppression.) Using these criteria, 321 liver recipients (86% of liver transplants carried out within the study time frame) and 368 renal recipients (74% of renal transplants within the time frame) were included in this study. The mean age of the liver transplant recipients was 48.9 years (range 19–83 years) with 108 females and 213 males. Among the renal transplant recipients, the mean age was 45 years (range 17–77) with 160 females and 208 males. All patients accepted onto the renal and liver transplant programs gave informed consent for their laboratory results to be analyzed for research purposes.

### Immunosuppression

Immunosuppression was according to in-house protocols. In the renal transplant patients, induction was with Basiliximab 20 mg IV on day zero and day 4. Patients were given 1g twice daily of mycophenolate mofetil (MMF) initially and reduced to 750 mg twice a day at a month (if no rejection) and 500 mg twice a day at 3 months. Tacrolimus was dosed on ideal body weight at 0.075 mg/kg twice daily aiming for initial levels of 10–12 ng/mL for the first month, 8–10 ng/mL for second month and 7–9 ng/mL for third month. All patients received a single dose of Methylprednisolone 500 mg at induction and oral/iv steroids to the equivalent of 20 mg were stopped at day 10 with some high-risk patients continuing to receive maintenance prednisolone 5 mg od thereafter.

In renal transplant patients with CMV DNAemia the initiation of ganciclovir or valganciclovir therapy was accompanied by a 50% reduction in the MMF dose and if DNAemia did not respond over the first week of therapy MMF was suspended.

In liver transplant patients, tacrolimus (Prograf, Fujisawa, Ltd., Killorglin, Ireland) at 0.1 mg/kg/day was given nasogastrically (within 6 h from LT) in two divided doses and started within 6 h after transplantation. Azathioprine was given intravenously and then orally (1 mg/kg/day), and methylprednisolone (16 mg/day intravenously) was given until oral intake was possible; then, 20 mg/day prednisolone was used. Tacrolimus dosing was evaluated every other day and was adjusted with the goal of maintaining a whole blood level of 5–10 ng/mL by microparticle enzyme immunoassay (ImxTacrolimus II, Abbott Laboratories, Abbott Park, IL, USA), particularly with poor renal and/or graft function. The azathioprine dose was not changed unless neutropenia developed. Prednisolone was gradually tapered from 3 weeks and then stopped between 3 and 6 months.

Acute cellular rejection episodes in the liver transplant patients was managed with pulsed methylprednisolone, three times 1 g/day and repeated if rejection continued (up to a maximum of 12 g in total) in addition to the protocol immunosuppression. If CMV DNAemia was detected in a liver transplant recipient, no changes in immunosuppressive therapy were undertaken initially. However, depending on immunological risk and/or severity of viremia (high-viral loads) tacrolimus levels were reduced at the discretion of the treating physician.

### CMV serostatus

The CMV IgG serostatus of all patients was determined pretransplant using Biomerieux VIDAS until July 2008 after which an Abbott Architect I2000 SR was used. Donor CMV IgG serostatus was determined using the same methods or, in the case of donors from other hospitals, was provided by the National Health Service British Transplant Service.

### CMV DNA surveillance and preemptive antiviral therapy

CMV DNA in whole blood was quantified using a real-time PCR approach described elsewhere (11). Whole blood samples for CMV surveillance were collected twice a week while patients remained in hospital and as out-patients for the first 60 days posttransplant, then once a week with a targeted minimum follow-up of the first 90 days after transplantation. Additional samples were collected from CMV viremic patients to follow episodes through to resolution. Post-90 days, whole blood samples for CMV PCR were obtained at every clinic visit or if CMV syndrome/disease was suspected.

Our previous natural history studies showed a median whole blood viral load of 175 500 genomes/mL in patients with CMV EOD (lower limit of the 95% confidence interval 37 000 genomes/mL; Ref. 12). Based on this, preemptive antiviral therapy (ganciclovir [5 mg/kg, bid] or valganciclovir [900 mg bid] with dose adjusted for renal function) was initiated when the viral load exceeded 3000 genomes/mL with the aim of preventing the viral load from reaching 37 000 genomes/mL, taking into account the average doubling-time of 1 day (13) and the twice-weekly timing of sampling. Therapy was discontinued following two consecutive samples where CMV DNA was undetectable (assay cut-off 200 genomes /mL). Previous work showed that changes in viral load were indistinguishable between patients treated with ganciclovir or valganciclovir (11), so we did not distinguish between use of these two drugs.

Among the renal transplant recipients with CMV viremia, the mean number of days of surveillance was 187 days (range 11–528 days) and among those without CMV viremia it was 162 days (range 6–335); for liver transplant recipients with viremia surveillance occurred over 149 days (range 5–415 days) and for those without, 94.9 days (range 0–247 days). Surveillance did not extend to 90 days in 65 patients due to early death posttransplant (n = 19), poor graft survival (n = 4) and poor compliance (n = 42).

### Definition of virological parameters

CMV viremia was defined as detection of CMV DNA in whole blood above the assay cut-off (200 genomes/mL). The duration of viremia was defined as the total number of days on which CMV DNA was detected, including repeated episodes of viremia in the same patient. A repeat episode of viremia was defined as the presence of CMV DNA in whole blood detectable following the resolution of a previous episode as documented by two consecutive negative samples. Doubling times of CMV during episodes of viremia and decline rates following therapy were calculated using the standard exponential function as previously described (13). Initially, the growth rate was determined based from the slope of virus load over time:


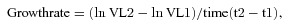


where VL2 is the viral load (genomes/mL) at time point t2 and VL1 is the viral load at time t1.

Doubling time (td) was then calculated using the equation:


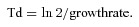


The D−R+ group was not included in this analysis as most viremic patients in this group were positive at only a single time point and liver and renal transplant recipients were combined to maximize group size. (Note that for completeness, we have not censored the subset of patients who move from <200 genome/mL to just above ∼250 and then resolve their viremia without treatment, thus the range for these values is broad.) The doubling times were used to calculate the basic reproductive number (R_0_; the average number of newly infected cells arising from a single infected cell when target cells are not limited) using standard formulae described previously (14,15).

CMV syndrome was defined as fever and leukopenia according to international guidelines (16). CMV EOD was defined using internationally agreed criteria including histological demonstration of inclusion bodies and/or positivity for CMV proteins by immunostaining, with the exception of CMV retinitis which was diagnosed ophthalmologically (17).

### Data collection

Demographic data on patients were collected from clinical databases. CMV viral load and histological data were collected from pathology databases.

### Statistical analyses

Comparisons of the proportion of patients who developed CMV viremia and association with clinical endpoints were performed using the chi-square test. The Mann–Whitney rank-sum test was used for all comparisons between the various donor/recipient (D/R) combinations within the transplant groups. The D’Agostino & Pearson omnibus K2 normality test (In Graph Pad Prism 5) was used for analysis of frequency distributions. Survival analysis was performed using Kaplan–Meier analysis and odds ratios for CMV viremia calculated using Cox proportional hazards models. All statistical analyses were performed using SAS v9.2 (SAS Institute Inc., Cary, NC, USA) unless otherwise stated. A two-sided p value ≤0.05 was deemed statistically significant.

## Results

### CMV viremia posttransplantation

Overall, 43% (294/689) of patients developed CMV viremia within the first 90 days posttransplant and 21% (142/689) required treatment because CMV viral load exceeded 3000 genomes/mL blood (Table 1). A further 5 (0.7%) liver and 16 (2.3%) renal transplant recipients developed a first episode of viremia after the first 90 days posttransplant. Thus, of the patients who developed viremia 294/315 (93.4%) did so within 90 days of transplantation. CMV syndrome developed in 36/689 (5.2%) patients whereas only 8/689 (1.2%) patients developed CMV EOD (Table 1). There were no significant differences between liver and renal transplant recipients in the proportion who developed CMV viremia (42% vs. 43%), who required treatment (20% vs. 22%) or who developed CMV syndrome or EOD (5.6% vs. 4.9%; 1.6% vs. 0.8%; Table 1).

**Table 1:** Outcomes in solid organ transplant patients managed using preemptive therapy

Transplant patient groups	Viremia^1^	Antiviral therapy	CMV syndrome	End-organ disease
Liver (n = 321)	136 (42%)	63 (20%)	18 (5.6%)	5 (1.6%)
Renal (n = 368)	158 (43%)	79 (22%)	18 (4.9%)	3 (0.8%)
Total (n = 689)	294 (43%)	142 (21%)	36 (5.2%)	8 (1.2%)

1 Onset within 90 days posttransplantation.

**Figure 1:**
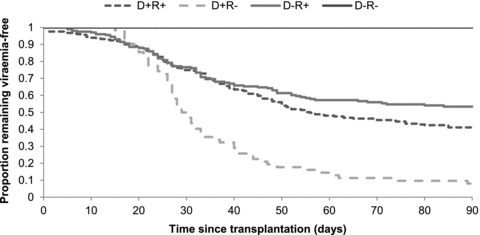
Kaplan–Meier survival analysis for CMV viremia, indicating the proportion of patients in each of the four DR groups remaining viremia-free through the 90 day follow-up period p < 0.0001 (log-rank test) for a comparison between the groups.

Analysis in the context of donor and recipient (D/R) CMV serostatus showed that, consistent with the established hierarchy of risk, the majority of D+R− patients developed viremia and required treatment (viremia: 58/74, [78%]; treatment: 51/74, [69%]). Indeed, D+R− status was the most significant risk factor for viremia (adjusted hazard ratio 3.56; 95% CI 2.49, 5.10; p < 0.0001; Table 2). For the D+R+ group 147/270 (54%) of patients developed viremia and 62/270 (23%) required treatment and for the D−R+ group 40% (89/222) developed viremia and 13% (29/222) required treatment. No D−R− patient developed viremia. A similar hierarchy among the groups was observed in the proportion of viremic patients requiring treatment: D+R− 51/58 treated (88%); D+R+ 62/147 (42%); D−R+ 29/89 (33%). D+R+ patients had an intermediate risk for viremia according to the Cox proportional hazards model (adjusted hazard ratio 1.30 [95% CI 1.0–1.68]; Table 2) though this increase was of borderline significance (p = 0.05) compared to the reference D−R+ group. Kaplan–Meier survival analysis of viremia (including those patients who did not complete 90 days of follow-up) revealed that, at 90 days posttransplant, 91.9% (95% CI: 85.5%, 98.7%) of D+R−, 58.9% (52.7%, 65.1%) of D+R+ and 46.64 (39.4%, 53.9%) of D−R+ patients had developed viremia (Figure 1).

**Table 2:** Results of Cox proportional hazards regression model; factors associated with time to development of CMV viremia

		Unadjusted			Adjusted		
			
		Hazard ratio	95% CI	p-Value	Hazard ratio	95% CI	p-Value
CMV donor/recipient status	D+R+	1.26	0.97, 1.63	0.08	1.30	1.00, 1.68	0.05
	D+R−	3.56	2.50, 5.08	<0.0001	3.56	2.49, 5.10	<0.0001
	D−R+	1.0 (reference)			1.0 (reference)		
Age at transplantation	Per 10 years	0.98	0.89, 1.07	0.22	1.00	0.91, 1.10	0.99
Type of transplant	Kidney	0.81	0.64, 1.02	0.07	0.81	0.64, 1.03	0.09
	Liver	1.0 (reference)			1.0 (reference)		

D−R− individuals are excluded from the analysis.

**Figure 2:**
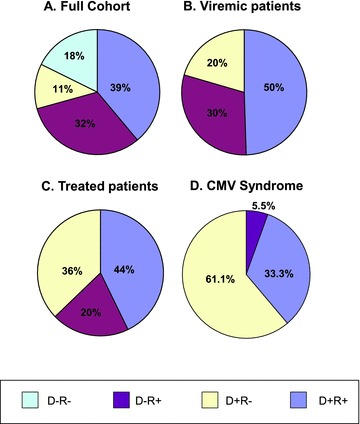
Pie charts illustrating the proportion of individuals in each DR group among (A) The total patient cohort; (B) those with viremia posttransplant, (C) those who received antiviral therapy; (D) those with CMV syndrome.

The D+R− group was the smallest of the four groups in terms of patient numbers (Figure 2A, Table 3), but it contributed a disproportionately large number of patients with CMV viremia, requiring treatment and with CMV syndrome (Figures 2B–D, Table 3). Indeed, the majority of CMV syndrome occurred within the D+R− group (Figure 2D, Table 3). However, in terms of absolute numbers, the majority of viremic and treated patients came from the two R+ groups, with the D+R+ group encompassing the largest proportion in each case (Figures 2B and C, Table 3).

**Table 3:** Viremia and treatment by donor:recipient serostatus

	D/R Group				
		
Patient group and category	D+R−	D+R+	D−R+	D−R−	Total^1^
Liver					
Number (% of total)	34 (11%)	112 (35%)	116 (36%)	59 (18%)	321
Viremic (% of DR group)	30 (88%)	64 (57%)	42 (36%)	0	136 (42%^1^)
Treated (% of DR group)	26 (76%)	27 (24%)	10 (9%)	0	63 (20%^1^)
% of viremics treated	87%	42%	24%	n/a	46%
CMV syndrome (% of DR group)	9 (26.5%)	8 (7.1%)	1 (0.9%)	0	18 (5.6%)
Renal					
Number (% of total)	40 (11%)	158 (43%)	106 (29%)	64 (17%)	368
Viremic (% of DR group)	28 (70%)	83 (53%)	47 (44%)	0	158 (43%^1^)
Treated (% of DR group)	25 (63%)	35 (22%)	19 (18%)	0	79 (21%^1^)
% of viremics treated	89%	42%	40%	n/a	50%
CMV syndrome (% of DR group)	13 (32.5%)	4 (2.5%)	1 (0.9%)	0	18 (4.9%)
Combined					
Number (% of total)	74 (11%)	270 (39%)	222 (32%)	123 (18%)	689
Viremic (% of DR group)	58 (78%)	147 (54%)	89 (40%)	0	294 (43%^1^)
Treated (% of DR group)	51 (69%)	62 (23%)	29 (13%)	0	142 (21%^1^)
% viremics treated	88%	42%	33%	n/a	48%
CMV syndrome (% of DR group)	22 (29.7%)	12 (4.4%)	2 (0.9%)	0	36 (5.2%)

1 All % values in “Total” column show % of total.

Taking the incidence of viremia in the D+R− group as a measure of the rate of transmission of virus from donor to recipient, CMV was transmitted with the donor liver in 30/31 (97%) patients who completed 90 days of follow-up, whereas after renal transplant the transmission frequency was lower at 70% (28/40).

A subset of patients experienced more than one episode of viremia. In the D+R− and D+R+ groups this occurred with a higher frequency in the renal group than the liver group (D+R− Renal: 20/28 [71%]; Liver 13/30 [43%], p = 0.02; D+R+ Renal: 50/83 [60%]; Liver: 25/64 [39%] p = 0.01).

### Virological parameters

#### Peak CMV load in donor/recipient groups

**Figure 3:**
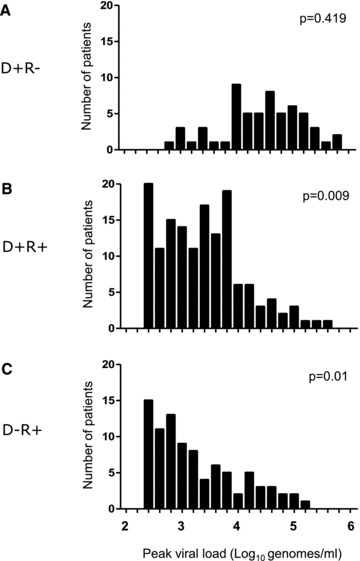
Frequency distribution plots of the values of peak viral load among the three DR groups of patients at risk of CMV infection Bin size is 0.2 Log 10 genomes. p-Values indicate difference from Gaussian distribution and were calculated with the D’Agostino & Pearson omnibus K2 normality test.

The peak viral load distributions for both groups of seropositive recipients were positively skewed, favoring lower peak values, whereas the values in the D+R− group more closely resembled a normal distribution (Figure 3). Interestingly, and consistent with previous observations (18), the D+R+ plot (Figure 3B) resembled a bimodal distribution reflective of a synthesis of the viral load profiles observed for primary infection (Figure 3A) and reactivation groups (Figure 3C).

**Figure 4:**
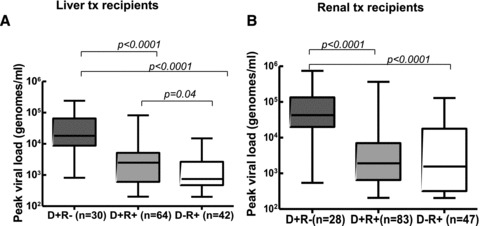
Peak viral loads in the three DR groups of liver (A) and renal (B) transplant patients at risk of CMV infections Line shows median value, box shows interquartile range and bars indicate range. p-Values are given only for significant differences.

For both liver (Figure 4A) and renal (Figure 4B) transplant patients, the median peak viral load was significantly higher in the D+R− group than in either of the R+ groups. (Liver: 18 165 genomes/mL [range 817–240 599] in D+R− patients versus 2484 [range 202–82 338] in D+R+ patients and 741 [range 200–14 968] in the D−R+ patients, p < 0.0001 for both comparisons; Renal: 42 901 genomes/mL [range 542–744 406] in D+R− patients versus 1908 [range 207–366 303] in D+R+ and 1551 [range 205–129 071] in D−R+; p < 0.0001for both comparisons). There was a minimal difference in median peak viral load between the D+R+ and the D−R+ recipients for either transplant group.

The median peak CMV viral load was marginally higher in the D+R− renal transplant recipients versus the liver D+R− transplant recipients (42 901 genome copies/mL vs. 18 165 genome copies/mL, p = 0.056), whereas there was no difference in median peak viral loads present in the D+R+ recipients between the transplant groups.

#### CMV replication kinetics

The rate of increase in CMV viral load in whole blood was significantly higher in the D+R− recipients with a median doubling time of 1.54 days (range 0.55–5.5) compared with 2.67 days (range 0.27–26.7) in the D+R+ recipients in the combined transplant groups (p < 0.0001). In addition, among D+R− recipients who developed a second episode of viremia, the median doubling time was longer than for the first episode (1.45 days for the first episode; 2.10 days for the second episode [p = 0.017]). A similar pattern was seen among the D+R+ recipients in the combined transplant group (2.17 days during the first episode and 2.82 days in the second episode [p = 0.023]).

Given previous estimates of the death rate of CMV infected cells (∼0.69/day; Ref. 14) we used the viral kinetics described above to estimate the basic reproductive number (R_0_) for CMV in these different settings. Median R_0_ values for the D+R− group was 2.02 (range 1.2–7.4) and for the D+R+ group was 1.48 (range 1.03–49.2). R_0_ values were lower for second viremic episodes: in the D+R− group, the median R_0_ value for the first episode was 2.11 and for the second, 1.67. For the D+R+ group the equivalent values were 1.63 and 1.45.

#### Duration of CMV viremia

**Figure 5:**
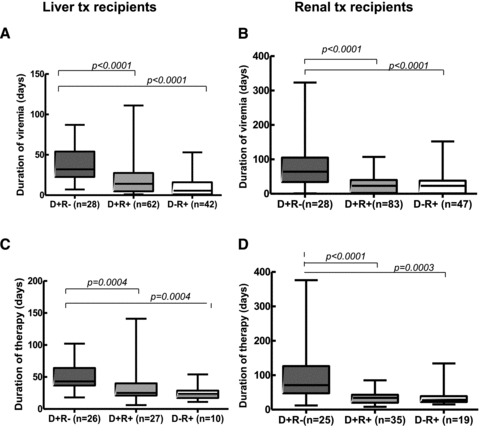
Duration of viremia (A, B) and duration of therapy (C, D) in the three DR groups of liver (A & C) and renal (B & D) transplant patients at risk of CMV infections Line shows median value, box shows interquartile range, and bars indicate range. p-Values are given only for significant differences.

The median total duration (all episodes combined) of CMV viremia in both liver and renal transplant patients was longer in D+R− patients compared to the D+R+ and D−R+. In the case of liver transplant recipients, the median values were 32 days (range 7–87), 14 days (range 1–111) and 5.5 days (range 1–53) for the D+R−, D+R+ and D−R+ groups (p < 0.0001; Figure 5A) whereas the corresponding values in the renal transplant patients were 64 days (range 1–323), 23 days (range 1–107) and 23 days (range 1–152), respectively (p < 0.0001; Figure 5B).

The median total duration of CMV viremia was significantly greater in the D+R− renal transplant recipients compared to the D+R− liver group (64 days vs. 32 days, p = 0.004) though there was no difference in total duration in the D+R+ recipients between the two transplant groups.

### Antiviral therapy

In the D+R− liver transplant patients, treatment was continued (duration of all treatment episodes combined) for a median of 43 days (range 18–102), compared to a median of 25 days (range 6–141) in D+R+ patients (p = 0.0004) and 23.5 days in the D−R+ patients (p = 0.0004; Figure 5C). A similar observation was made for the D+R− renal transplant recipients, where the total duration of treatment was significantly longer (median of 71 days; range 12–376) compared to the D+R+ patients of 34 days (range 8–85; p < 0.0001) and the D−R+ patients of 27 days (range 15–134), p = 0.0003 (Figure 5D).

The median total duration of anti-CMV treatment required in the D+R− renal transplant group was significantly greater than in the equivalent liver group (71 days vs. 43 days, p = 0.006), with no significant difference in total duration of treatment required among the D+R+ recipients of either transplant group. After adjusting for the type of organ transplanted and D/R status, the total number of days of anti-CMV therapy was strongly associated with peak viral load. Each 1 log higher peak viral load was associated with 31 days (95% CI 19–43 days) longer total treatment time (p = 0.0001).

We also calculated the proportion of viremic patients in each group that required therapy (Table 3). For the D+R− and D+R+ groups this was similar for renal and liver patients, but for the D−R+ group, 24% (10/42) of viremic liver patients required therapy, whereas for renal patients the equivalent value was 40% (19/47). All patients with viral loads >3000 genome copies/mL blood cleared their CMV viremia with treatment indicating that clinical drug resistance to CMV was not problematic in these patients.

### CMV viremia and rejection

CMV viremia was more common in patients who had experienced graft rejection (managed with increased immunosuppression) within the first 3 months postliver transplant than those who did not experience rejection (viremia was detected in 71/131 [54%] of patients with rejection and 69/174 [40%] patients without rejection p = 0.006.) Notably, biopsy-proven rejection was reported before the detection of CMV DNA in whole blood in 67 of the 71 patients (94%). In the liver group, there were 70 individuals on whom data on date of viremia and date of rejection were available. Rejection occurred after the occurrence of viremia in 3/70 cases (4.3%; 1 day prior in 2 cases and 6 days prior in 1 case). In this group, the median times from transplantation to rejection and occurrence of viremia were 7 (range 3–25) days and 23 (range 5–85) days, respectively. The median time between transplantation and rejection was 14 (range 6–76) days.

Among renal transplant recipients, biopsy proven rejection was observed in an equal number of viremic (21/158; 13%) and nonviremic (19/210; 9%) patients within the first 3 months posttransplant. Again, rejection was detected before onset of viremia in the majority (17/21, 81%) of cases. The median number of days from transplant until rejection did not differ significantly with CMV status in either transplant group (Liver: viremic: median 7 days, range 3–25 days; nonviremic: 7 days, range 3–34 days. Renal: viremic: 12 days, range 3–111 days; nonviremic: 8 days, range 3–83 days). There was no difference in the 1-year patient survival rates observed in the viremic and nonviremic recipients in either transplant group.

## Discussion

This comprehensive analysis of a large prospectively followed solid-organ transplant cohort managed by quantitative PCR driven preemptive therapy allowed us to critically evaluate whether the historic indicators of risk of CMV infection and associated kinetics of replication were still evident. Although early antiviral intervention minimizes the risk of progression to high-level viral load and CMV disease, D+R− patients continue to have significantly faster viral replication kinetics and higher peak viral loads despite having very low levels of EOD. The occurrence of CMV syndrome was most evident in the D+R− group and in the renal patients was observed at a similar frequency (32.5%) to that observed following the cessation of prophylaxis (4).

Although many studies tend not to differentiate between renal and liver transplant recipients, our analysis revealed that from a CMV perspective some important differences exist between these two patient groups. For example, the D+R− liver group experienced almost universal CMV infection whereas only 70% (28/40) of the comparable group of renal patients experienced CMV viremia. Peak CMV load, however, was greatest in the D+R− renal transplant recipients even though the doubling time of CMV was comparable between the two transplant groups. D+R− renal transplant recipients consequently required a longer total period of antiviral therapy and, interestingly, were more likely to suffer a second viremic episode. Other studies have shown that the latter is related to CMV replication kinetics and a slow response to therapy and this is consistent with our observations (19). Indeed, our data suggest that a hybrid management approach should be formally evaluated for high-risk renal patients, in which patients are managed preemptively until resolution of the first viremic episode, at which point delayed prophylaxis is commenced.

Overall, our data emphasize that a subset of D+R− patients control their viremia without antiviral therapy or avoid it entirely. Thus, 30% of the D+R− patients in our cohort, all of whom would have received extended ganciclovir therapy if managed prohylactically, did not receive any antiviral therapy. Other studies have not observed this effect (20) which reflect the different immunosuppressive regimens deployed, or delays in transplantation and cold ischemia times which affect cell viability.

The higher proportion of renal (40%) than liver (24%) viremic D−R+ patients requiring treatment in our cohort suggests that the former were comparatively less able to control CMV reactivation. This observation may reflect the greater burden of immunosuppression, including the universal use of anti-CD25 antibodies, in the renal transplant group minimizing the effectiveness of the host immune response (21).

More broadly these observations document, albeit indirectly, likely differences in the impact of preexisting CMV specific immunity on control of viremia between the two transplant groups. We and others have demonstrated quantitative differences in frequency and quality of T-cell responses observed in liver and renal transplant patients (21–25) and this study emphasizes the important differences in immune control of CMV in different patient groups. Our virological analyses provide an assessment of the quantitative effects of preexisting immunity on virus replication. This is most striking in reduction in R_0_ in R+ compared to D+R− patients (2.02 reduced to 1.48). Interestingly, a reduction of similar magnitude was observed between the first and second episodes of replication in the D+R− group (R_0_ 2.11 reduced to 1.63). These relatively low R_0_ values in a recent cohort of patients contrast with earlier R_0_ estimates in liver transplant patients (14) and emphasizes the potential for controlling CMV replication by immunization (10). Regardless, these values provide an integrated virological parameter to incorporate into the design of future pharmacodynamic assessments of antiviral drugs and prototype CMV vaccines.

In summary, our study has revealed some important differences in the natural history of CMV infection between liver and renal transplant recipients in the era of aggressive preemptive therapy. Our results provide important quantitative information which can be used to design and power future studies of vaccines and/or antiviral drugs with activity against CMV.

## Abbreviations

CMV: cytomegalovirus
qPCR: quantitative real-time polymerase chain reaction
Ro: basic reproductive number
SOT: solid organ transplant
